# The inhibitory effects of an eight-herb formula (RCM-107) on pancreatic lipase: enzymatic, *HPTLC* profiling and *in silico* approaches

**DOI:** 10.1016/j.heliyon.2019.e02453

**Published:** 2019-09-12

**Authors:** Shiqi Luo, Harsharn Gill, Daniel Anthony Dias, Mingdi Li, Andrew Hung, Linh Toan Nguyen, George Binh Lenon

**Affiliations:** a School of Health and Biomedical Sciences, RMIT University, Bundoora West Campus, Victoria, 3083, Australia; b School of Science, RMIT University, Bundoora West Campus, Victoria, 3083, Australia; c Department of Endocrine, 103 Military Hospital, Vietnam Military Medical University, Hanoi, Viet Nam

**Keywords:** Analytical chemistry, Computational chemistry, Molecular biology, Alternative medicine, Evidence-based medicine, Obesity, Traditional Chinese medicine, Lipase inhibition, Catechins, High-performance thin layer chromatography (HPTLC), Docking

## Abstract

**Aims:**

Obesity is a global, public health issue that causes or exacerbates serious medical disorders. Chinese herbal therapies have become one of the most popular alternatives due to intolerances of current anti-obesity treatments. The RCM-107 formula (granule) is modified from our previous studied RCM-104 formula, which has demonstrated significant effects on weight reduction in randomized clinical trials. Up to date, there is no published scientific evidence to evaluate the effect of this formula on the weight-loss target pancreatic lipase and therefore, the aim of this study is to investigate the inhibitory effect of RCM-107 and respective individual ingredient on the pancreatic lipase activities.

**Main methods:**

Fluorometric based enzymatic assays, high-performance thin-layer chromatography (HPTLC) profiling and *in silico* molecular docking techniques were used to investigate the lipase inhibitory effects of the RCM-107 herbal formula and its respective individual herbs.

**Principle findings:**

The results demonstrated the potent lipase suppressing effect of the RCM-107 formula. The majority of the ingredients from this formula also showed pancreatic lipase inhibitory activities. The presence of the known weight-loss compounds such as (-)-epigallocatechin-3-gallate (EGCG), epicatechin-3-gallate (ECG), (-)-epicatechin (EC), rutin, crocin and caffeine were identified in the RCM-107 and related single herbs using HPTLC profiling approaches. In addition, EGCG, EC and the known lipase antagonist orlistat acted on the same site. These compounds form hydrogen bonds with corresponding residues HIS152, ASP80 and GLY77, which can be considered as markers of important areas in the ligand-binding site. This may explain the details of their roles in inhibiting pancreatic lipase activities.

**Conclusion:**

Our data has provided new knowledge to the mechanistic properties of the RCM-107 formula and its respective individual herbal ingredients for weight loss, in terms of reducing lipid absorption via the inhibition of pancreatic lipase.

## 1 Introduction

Obesity is a worldwide epidemic leading to disorders such as hypertension, Type 2 diabetes, cardiovascular diseases and non-alcoholic fatty liver disease (Xu et al., 2015). At least 2.8 million adults die due to being overweight or obese each year (World Health Organization (WHO), 2019). Globally, over 1.9 billion adults were overweight while more than 650 million adults were obese in 2016 (World Health Organization (WHO), 2019). In Australia, a total of 67% of adults were overweight, in which 31.3% were obese in 2017–2018 (Australian Bureau of Statistics (ABS), 2018).

A promising strategy for weight reduction is to suppress nutrient digestion and absorption (Buchholz and Melzig, 2015; Rodgers et al., 2012; Sun et al., 2016). Pancreatic lipase (PL), a known enzyme playing a crucial role in lipolysis, promotes fatty acids absorption in the small intestine (Buchholz and Melzig, 2015; Luo et al., 2019). Orlistat is a known effective pancreatic lipase inhibitor that has been used for the long-term weight management since 1999 (Luo et al., 2019; Rodgers et al., 2012). However, it can lead to gastrointestinal intolerances, including faecal urgency and faecal incontinence (Chen, 2016; Lucas and Kaplan-Machlis, 2001; Rodgers et al., 2012). Hence, greater attention has been drawn to herbal medicine due to their potentials for offering novel, weight-loss treatments. However, scientific validation is required to validate these claims for the use of these popular products (Marrelli et al., 2014).

The RCM-107 formula (granule) has been modified from our previous studied RCM-104 formula which demonstrated significant weight reduction in the clinical trial (Lenon et al., 2012) by adding additional herbs known to contain weight-loss bioactive compounds. This formula contains eight Chinese herbs, including *Camellia sinensis* (green tea), *Poria*, *Nelumbinis folium* (lotus leaf), *Alismatis rhizoma*, *Plantaginis semen*, *Cassiae semen*, *Sophorae flos* and *Gardeniae fructus*. The active components for weight reduction or lipase inhibition from the individual crude herbs presenting in the RCM-107 formula have been previously described in the scientific literature. Tea catechins such as EGCG, ECG, EC and (+)-catechin have been reported to play important roles in weight reduction (Nagle et al., 2006). For example, Grove et al. (2012) studied the pancreatic lipase inhibition effects of EGCG, which is the most active and abundant compound found in green tea (Chakrawarti et al., 2016; Nagle et al., 2006). Caffeine from green tea can reduce weight by increasing energy expenditure *via* promoting lipid oxidation and thermogenesis (Quinhoneiro et al., 2018).

In addition, Miao et al. (2017) indicated that *Alismatis rhizoma* contains triterpenoids (such as alisol B acetate) as the main bioactive compound that can reduce serum total cholesterol and triglycerides levels. Anthraquinones, in particular, chrysophanol (Locatelli, 2011), has been considered as one of the main bioactive ingredients present in *Cassia semen* that may be responsible for its lipid-lowering activities (Awasthi et al., 2015). In a study by Sheng et al. (2006), the authors found that crocin present in *Gardeniae fructus* can be used as an effective anti-hyperlipidemic agent as it could interfere with pancreatic lipase activity and contribute to the reduction in lipid absorption. Furthermore, Ahn et al. (2013) and Ha do et al. (2010) suggested that flavonoids such as quercetin and rutin from lotus leaves and *Sophorae flos* are beneficial for suppressing fat accumulation. Quercetin-3-*O*-D-glucuronide (Q3*O*G), is one of the flavonoids in lotus leaves and has also shown strong inhibitory effects of porcine pancreatic lipase (Ahn et al., 2013).

To our best knowledge, there has been no published research investigated the effects of the RCM-107 formula on the weight-loss target pancreatic lipase. In this study, we aim to explore the lipase inhibitory activities of the modified herbal formula and respective individual herbs, as well as identify the presence of the bioactive weight-loss compounds mentioned earlier in the formula. The interactions between potential lipase inhibitors (chemical compounds) to the target enzyme (pancreatic lipase) were determined to identify likely mechanistic interactions for ligand-target binding.

## 2 Methods

### 2.1 Materials

Lipase from porcine pancreas (type VI-S L0382-100KU), orlistat (04139-25 mg) and 4-methylumbelliferyl oleate (4-MUO 75164-25 mg) were obtained from Sigma-Aldrich Australia. Chemical references EGCG, ECG, EC, caffeine, rutin, crocin, solvents used for high-performance thin layer chromatography (HPTLC) mobile phase (i.e. toluene, acetone, formic acid, ethyl acetate, glacial acetic acid, chloroform, methanol and ethanol), derivatization reagents: Fast Blue Salt B, Natural Products Reagent (NP reagent), Polyethylene glycol (PEG solution), 10% sulphuric acid reagent and *p*-anisaldehyde-sulfuric acid reagent were also purchased from Sigma-Aldrich, Australia. The RCM-107 capsules (AUST L 285569) were obtained from Tong Kang Lee Chinese medicine clinic and single herbal granules (extracted products) of Fu ling/*Poria*, He Ye/lotus leaf, Ze Xie/*Alismatis rhizoma*, Che Qian Zi/*Plantaginis semen*, Jue Ming Zi/*Cassiae semen*, Huai Hua/*Sophorae flos*, Zhi Zi/*Gardeniae fructus*, green tea (matcha) were supplied by GL natural health care Chinese medicine clinic. HPTLC glass plates 20 × 10 cm coated with silica gel 60 F_254_ were produced by Merck. Chromatographic equipment (Linomat 5 sample applicator; ADC 2 developing chamber; automated derivatizer; visualizer; VisionCATS 2.4 software) were supplied by CAMAG. Molecular docking was conducted via virtual screening software PyRx (Version 0.8).

### 2.2 Lipase inhibition assay

#### 2.2.1 Herbal extraction

Individual capsule of RCM-107 containing 500 mg herbal powder was mixed with starch. All concentrated herbal powders and granules were obtained by water extraction of the raw materials via boiling and lyophilisation performed by the manufacturer. Twenty milligrams of the RCM-107 herbal powder and the eight individual herbal granules were weighed and dissolved in 4 mL of Milli-Q® Water (Merck Millipore Milli-Q® Integral Water Purification System) mixed with 2% dimethyl sulfoxide (DMSO). The samples were subsequently vortexed and sonicated for 10 min then microfiltered through a Millex-HP 0.45μm filter (Millipore). Dilution from the stock solution (5 mg/mL) was performed to achieve a final concentration of 0.1 mg/mL. In addition, serial dilution of the RCM-107 formula was performed to obtain seven point calibration of this sample (0.001–0.3 mg/mL).

#### 2.2.2 Measurement of pancreatic lipase activity

The fluorometric assay of pancreatic lipase activity using 4-MUO as a substrate was carried out according to the modified methods previously described by Nakai et al. (2005) and Podsedek et al. (2014). Twenty-five microliters of the RCM-107 formula sample solution (0.001–0.3 mg/mL), each single herbal granule (0.1 mg/mL), 25 μL of the lipase solution (50 U/mL) were prepared in buffer (13 mM Tris-HCl, 75 mM NaCl 1.3 mM CaCl_2_ pH 8.0) and pre-incubated at room temperature for 5 min. To initiate the enzyme reaction, 50 μL of 0.1 mM 4-MUO solution was dissolved in the same buffer as described above and was added to the pre-incubation mixture followed by incubation for an additional 30 min at room temperature. The amount of 4-methylumbelliferone produced was measured with a *CLARIOstar*® microplate reader (BMG labtech) using an excitation and emission wavelength of 355 nm and 460 nm, respectively. Orlistat (a pancreatic lipase inhibitor) was included in each test as a positive control. Wells that contained either samples and buffer or enzyme and buffer were used to detect background autofluorescence. The inhibition activity (%) of the pancreatic lipase was calculated using the following equation:

1-(F_sample_-F_sample background_-F_blank_) / (F_control_-F_control background_-F _blank_)*100

F_sample_ and F_sample background_ represent fluorescence values of the sample solution with or without substrate, while F_control_ and F_control background_ are fluorescence values of the control (no inhibitors) with or without substrate, respectively. F_blank_ is the fluorescence value of the chosen blank, which consists of substrate and buffers only.

#### 2.2.3 Statistical analysis

Samples and controls were all run in triplicates. Results were presented as mean ± SEM from three independent experiments. The concentration providing 50% inhibition (IC_50_) was calculated by non-linear regression (values are mean ± SD) and statistical significance was assessed with one-way analysis of variance (ANOVA) followed by the Tukey multiple comparison tests via GraphPad Prism 7 software. Results with *P* < 0.05 have been considered statistically significant.

### 2.3 High-performance thin-layer chromatography

#### 2.3.1 Sample and standard extraction

The RCM-107 formula (0.5 g) was individually mixed with 10 mL of either ethanol; methanol; ethanol-water 8:2, methanol-water 8:2 and ethyl acetate. Single granules such as green tea powder (0.4 g) were mixed with 4 mL ethanol while lotus leaf granules (0.4 g) were mixed with 4 mL methanol. Granules of *Cassia semen* (1 g), *Gardeniae fructus* (1 g), *Sophorae flos* (1 g) were prepared in 10 mL of methanol and *Alismatis rhizoma* (1 g) was dissolved in 10 mL of ethyl acetate. All samples were vortex-mixed for 2 min and centrifuged at 4000 rpm for 10 min. The supernatants were obtained for HPTLC profiling.

Chemical standards of EGCG, ECG, EC and caffeine were individually dissolved in methanol to prepare stock solutions and subsequently, serially diluted to achieve a final concentration of 10 μg/mL. Quercetin, rutin and Q3*O*G were prepared in ethyl acetate individually prepared stock solutions and subsequently, serially diluted to make a final concentration of 100 μg/mL. Crocin was mixed in a 1:1 methanol-water to obtain a stock solution (1 mg/mL) and subsequently, serially diluted to 100 μg/mL. Chrysophanol and alisol B acetate were prepared in ethyl acetate to prepare stock solutions and subsequently, serially diluted to yield a final concentration of 10 μg/mL and 100 μg/mL, respectively.

#### 2.3.2 Chromatography

Herbal samples (2 μL) and reference standards (5–20 μL) were applied to the plates as 6–8 mm bands, 7.8–11.4mm apart via Linomat 5 sample applicator from CAMAG. Dosage speed varied from 75 nL/s-150 nL/s depending on the type of solvents used. Plates were developed to a distance of 70 mm in the pre-saturated (25 min) and pre-drying was carried out in an ADC 2 developing chamber (a twin trough chamber) (Reich et al., 2006).

#### 2.3.3 Derivatization

Polyphenols (EGCG, ECG and EC): 2.0 mL of Fast Blue Salt B reagent was sprayed over the plate. Evaluation was performed under white light or UV 254 nm (Schibli and Reich, 2005). Flavonoids (quercetin, rutin and Q3*O*G): 2.0 mL of NP reagent was sprayed over the plate, with a 5 min delay, then 2.0 mL of PEG solution was sprayed. Evaluation was performed under UV 366 nm (Schibli and Reich, 2005). Crocin: no derivatization was required. Evaluation was performed under white light after development (Wagner et al., 2016). Anthraquinones (chrysophanol): 2.0 mL of 10% sulphuric acid reagent was sprayed over the plate, followed by heating of the plate at 100 ^ͦ^ C for 3 min. Evaluation was performed under UV 366 nm (Priya et al., 2013). Triterpenoid (alisol B acetate): 2.0 mL of *p*-anisaldehyde-sulfuric acid reagent was sprayed over the plate, and the plate was heated at 100 ^ͦ^ C for 3 min, followed by evaluation under white light (Wagner et al., 2016).

#### 2.3.4 Documentation

Images of the plates were taken before or after development and after derivatization under white light, UV254 nm and UV366 nm via the visualizer. Reports were generated using Vision CATS 2.4 software (Reich et al., 2006).

#### 2.3.5 Compound identification

HPTLC profiling was used as a rapid tool purely to efficiently identify the nominated compounds by comparing the system suitability parameter (*Rf* values) of the authentic standards to that either present or absent in the tested sample (Kumar et al., 2008; Renger et al., 2011). In all cases, the mobile phases were optimized prior to chromatographic separation (Kumar et al., 2008).

The herbal samples were extracted using different solvents to ensure the efficient extraction of compounds from the samples. The experiments were repeated in six independent analyses to ensure accuracy and repeatability (Kumar et al., 2008).

### 2.4 Molecular docking

The published three-dimensional (3D) structure of pancreatic lipase (PDB code:1ETH, a dimer) was acquired from the RCSB protein databank, which was modified using the program Visual Molecular Dynamics (VMD) to obtain protein-only structures (Dallakyan and Olson, 2015). Structures of the weight-loss or lipid inhibiting compounds presented in the HPTLC experiments were downloaded from PubChem (https://pubchem.ncbi.nlm.nih.gov/). Both selected protein and ligand files were loaded to PyRx as macromolecules and ligand, respectively. The reference protein and ligands were placed inside an enclosed box with ‘centre’ X: 8.3130; Y: 73.5614; Z: 146.1870 and ‘dimensions’ X: 58.2669; Y: 72.8369; Z: 57.5022, followed by docking process initiated via the AutoDock Vina from PyRx. The exhaustiveness was set as 64. The binding affinity scores were recorded using Microsoft Excel. Two-dimensional (2D) and 3D interactions between the ligands and protein were observed from Discovery Studio Visualizer (DSV).

## 3 Results

### 3.1 In vitro inhibition of pancreatic lipase

**Fig. 1:**
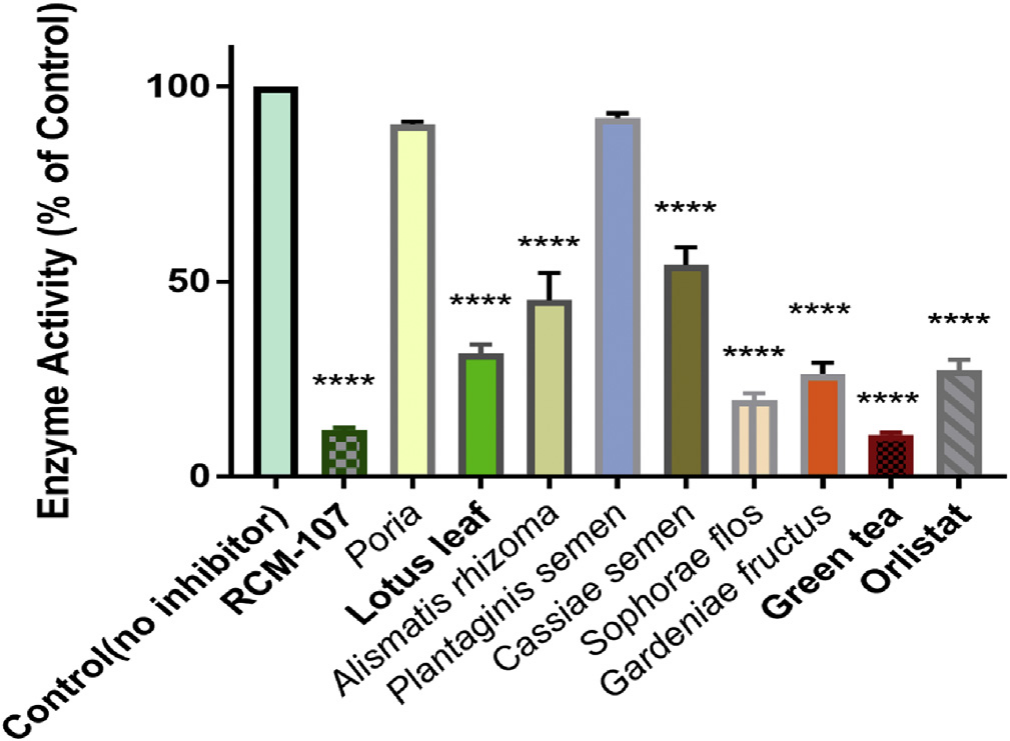
Suppressive effects of the RCM-107 formula, eight single herbal granules and orlistat (inhibitor) at 100 μg mL^−1^ on pancreatic lipase activity. The lipase activity with an absence of the samples or inhibitor was presented as 100%. Data is expressed as means ±SEM from three independent experiments, including three replicates each time. **** indicates *P* < 0.0001 as compared to the control, R square: 0.983.

The effects of the RCM-107 formula, 8 single herbal granules present in the modified formula included *Poria*, lotus leaf, *Alismatis rhizoma*, *Plantaginis semen*, *Cassiae semen*, *Sophorae flos*, *Gardeniae fructus*, green tea and the positive control orlistat on porcine pancreatic lipase were examined (Fig. 1). All samples were screened at a concentration of 100 μg/mL and lipase activity was presented as a percentage of the control (with no inhibitor). Four herbal samples displayed potent inhibitory effects than the known inhibitor, orlistat whilst green tea exhibited the highest inhibition rate of 89 % which was slightly but non-significantly higher than that of the RCM-107 formula (88 %) (*P* > 0.05). *Sophorae flos* and *Gardeniae fructus* displayed an average inhibition level of 80 % and 74 %, respectively whereas orlistat showed a 73% inhibition of lipase. The differences in inhibition between green tea, the RCM-107 formula and orlistat were considered as statistically significant (*P* < 0.05).

Lipase inhibitors which showed minimal potency were also identified. Lotus leaf, *Alismatis rhizoma* and *Cassiae semen* significantly reduced lipase activity with inhibition rates of 68 %, 55 % and 46 %, respectively (*P* < 0.0001). The two herbal granules *Poria* and *Plantaginis semen* were found to display mild inhibitory activities (10% and 8%) compared to the control, however, the differences were statistically insignificant (*P* > 0.05).

**Fig. 2:**
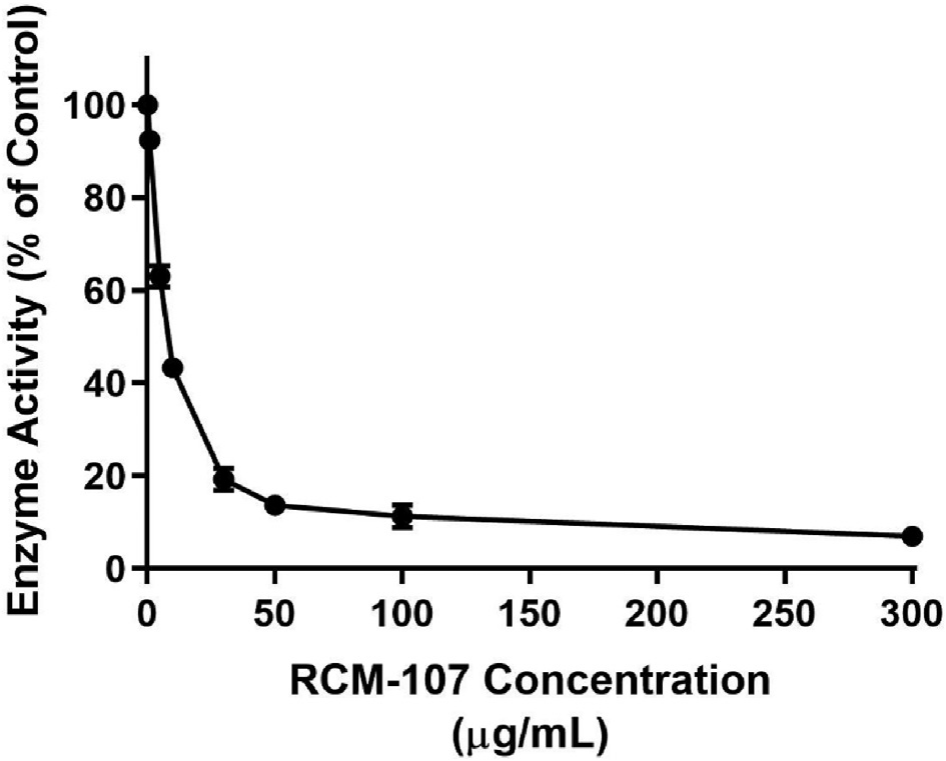
Dose-dependent inhibitory effects of the RCM-107 formula (1-300 μg mL^−1^) on porcine pancreatic lipase. Data represent mean ± SEM from three independent experiments with three replicates per condition.

Additionally, the RCM-107 formula was assayed using a seven-point calibration curve, which displayed inhibitory effects in a dose-dependent manner (Fig. 2). This herbal formula presented potent pancreatic lipase inhibitory activities with an IC_50_ value of 7.17 ± 0.69 μg/mL (mean ± SD). The enzyme activity was dramatically suppressed when the concentration of the RCM-107 formula increased from 1 μg/mL to 300 μg/mL, which exhibited an inhibition rate from 8% to 93%, respectively.

### 3.2 HPTLC

#### 3.2.1 Method specificity

Authentic standards at different concentrations were applied in each chromatography plate, including 0.05, 0.1, 0.15, 0.2 μg/spot of EGCG, ECG, EC, caffeine and chrysophanol; 0.5, 1, 1.5 μg/spot of quercetin, rutin, Q3*O*G and crocin; 0.5, 1, 1.5, 2 μg/spot alisol B acetate. Optimized concentrations of herbal samples were carried out in all experiments, including 0.1 mg/spot of RCM-107 formula; 0.2 mg/spot of green tea; lotus leaf; *Cassia semen*; *Gardeniae fructus***;** *Sophorae flos* and *Alismatis rhizome*.

Varying compositions and ratios of toluene, acetone, formic acid for separating caffeine and polyphenols such as EGCG, ECG, EC; ethyl acetate, formic acid, glacial acetic acid, water for flavonoids like quercetin, rutin and Q3*O*G; chloroform, methanol, water for crocin; ethyl acetate, methanol, water for chrysophanol; chloroform, methanol, water for alisol B acetate were examined. The optimized forms of mobile phase were adjusted and used in the experiments.

#### 3.2.2 Identification of EGCG, ECG, EC and caffeine

**Fig. 3:**
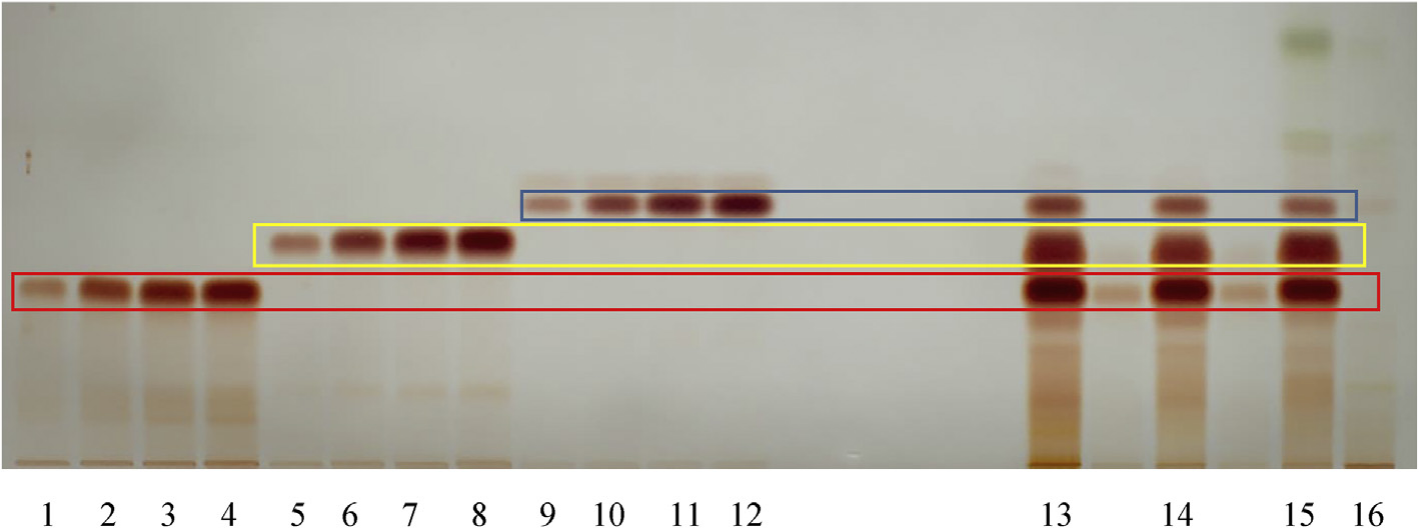
HPTLC fingerprint (polyphenols) of herbal samples under white light after derivatization. 1–12: chemical reference substances: EGCG, ECG, EC in different volumes (5–20 μL), respectively; 13: the RCM-107 formula in methanol; 14: the RCM-107 formula in ethanol; 15: green tea sample; 16: Lotus leaf sample.

**Fig. 4:**
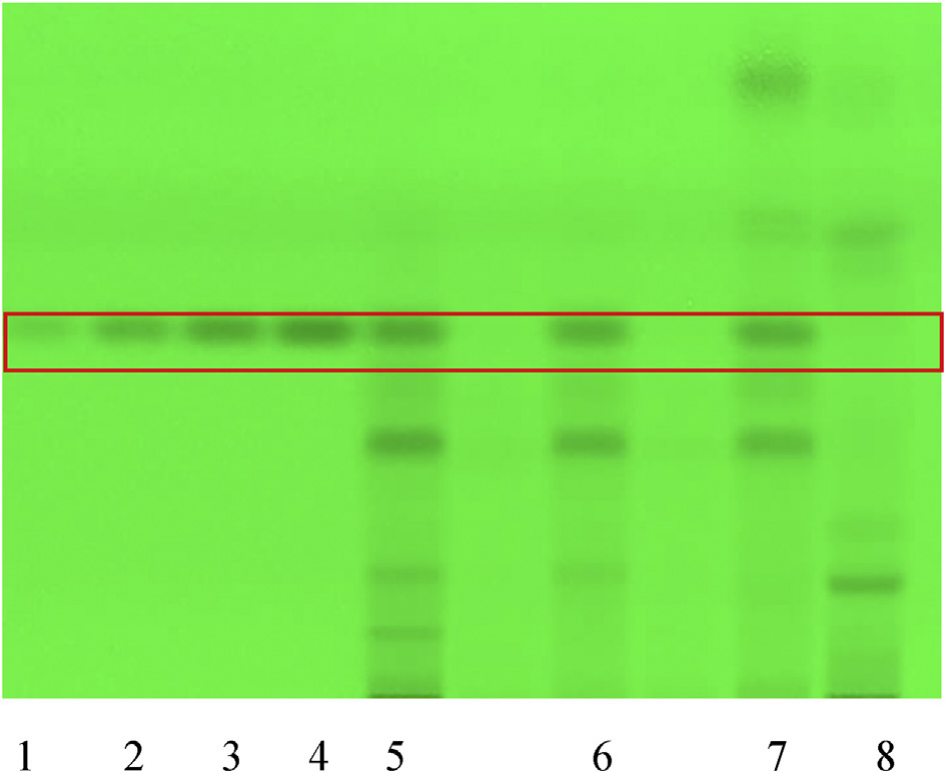
HPTLC fingerprint (caffeine) of herbal samples after development under UV 254 nm. 1–4: chemical reference caffeine in different volumes (5–20 μL); 5: the RCM-107 formula in methanol; 6: the RCM-107 formula in ethanol; 7: green tea sample; 8: Lotus leaf sample.

HPTLC fingerprints were generated for the RCM-107 formula and green tea samples, which were developed on silica gel using the mobile phase toluene, acetone, formic acid 9:9:2 (v/v/v) (Schibli and Reich, 2005). After derivatization with Fast Blue Salt B reagent (Schibli and Reich, 2005), all polyphenols were readily identified under white light. EGCG, ECG and EC with increasing *Rf* values (0.35, 0.46 and 0.54, respectively) were used as authentic standards (Fig. 3). In addition, caffeine displayed an *Rf* value of 0.523 under 254 nm (UV light) after development but without derivatization (Fig. 4). All four polyphenols were identified in the RCM-107 formula and green tea samples, except in the lotus leaf sample by comparison of the *Rf* of the authentic standard to that identified in the samples.

#### 3.2.3 Identification of quercetin, rutin and Q3*OG*

**Fig. 5:**
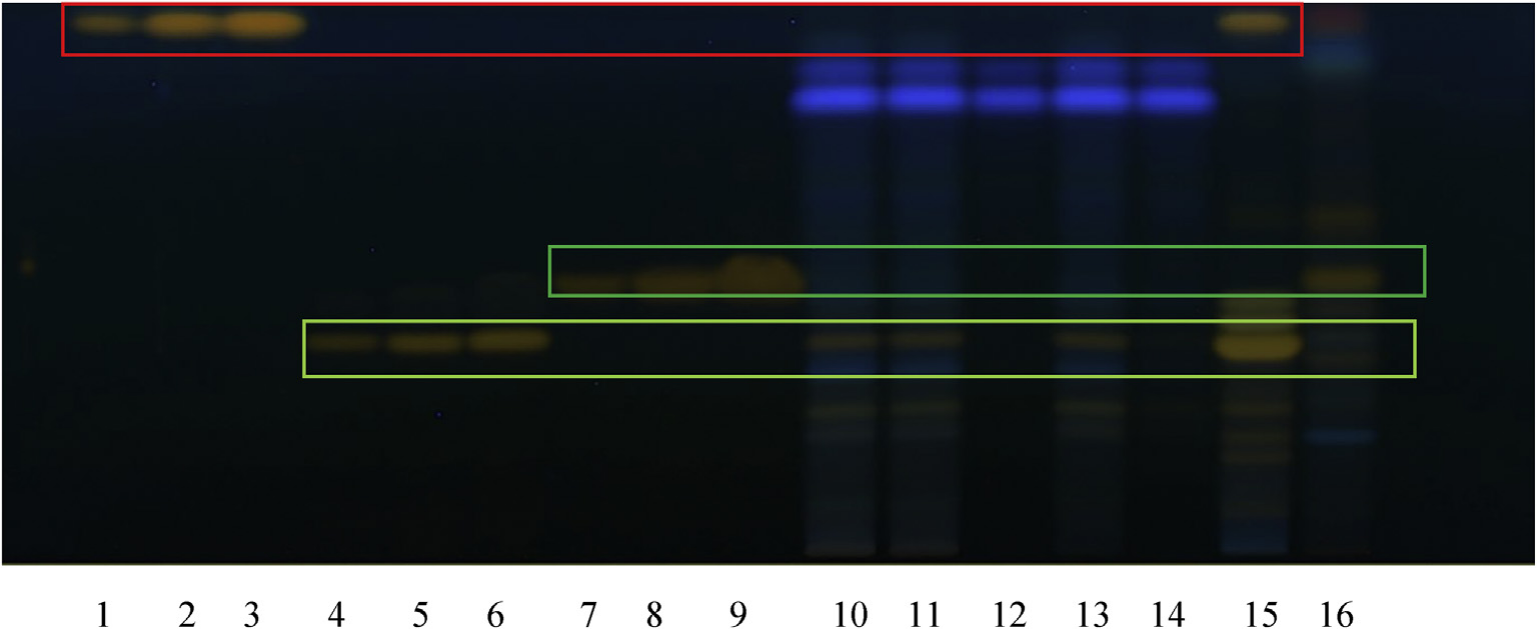
1–3: reference substance: quercetin (5–15μL); 4–5: reference substance:rutin (5–15μL); 7–9: reference substance: quercetin-3-*O*-D-glucuronide (5–15μL); 10: the RCM-107 formula in methanol-water 8:2; 11: the RCM-107 formula in ethanol-water 8:2; 12: the RCM-107 formula in ethyl acetate; 13: the RCM-107 formula in methanol; 14: the RCM-107 formula in ethanol; 15:*Sophorae flos* sample; 16: Lotus leaf sample.

HPTLC fingerprints of flavonoids were identified in the RCM-107 formula, *Sophorae flos* and lotus leaf samples which were developed on silica gel using the mobile phase ethyl acetate, formic acid, glacial acetic acid, water 100:11:11:27 (v/v/v) (Schibli and Reich, 2005). Following chemical derivatization with NP reagent first followed by the PEG solution (Schibli and Reich, 2005), all 3 flavonoids were observed under 366 nm quercetin, rutin and Q3*O*G with increasing *Rf* values (0.96, 0.41 and 0.52, respectively) were used as authentic standards (Fig. 5). Only rutin was identified to be present in the RCM-107 formula. Both quercetin and rutin could be identified in the *Sophorae flos* sample while Q3*O*G was identified exclusively in the lotus leaf sample by comparison of the *Rf* of the authentic standard to that identified in the samples.

#### 3.2.4 Identification of crocin

**Fig. 6:**
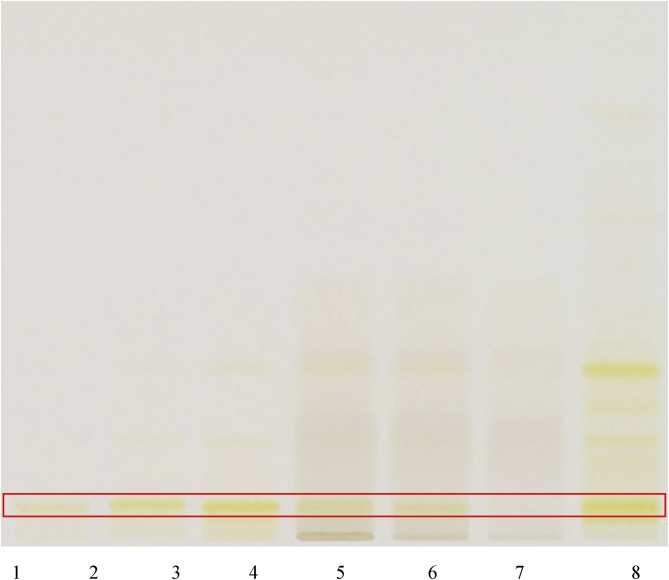
1–3: reference substance Crocin (5–15μL); 5: the RCM-107 formula in methanol-water 8:2; 6: the RCM-107 formula in methanol; 7: the RCM-107 formula in ethanol; 8: *Gardeniae fructus* samples.

HPTLC fingerprints of crocin were identified in the RCM-107 formula and *Gardeniae fructus* sample which were developed on silica gel using the mobile phase chloroform, methanol, water 70:30:4 (v/v/v) (Wagner et al., 2016). No derivatization was required, and results were observed under white light. Crocin gave an *Rf* value of 0.1 as the authentic standard (Fig. 6). Crocin was found to be present in the RCM-107 formula and *Gardeniae fructus* samples by comparison of the *Rf* of the authentic standard to that identified in the samples.

#### 3.2.5 Identification of chrysophanol

**Fig. 7:**
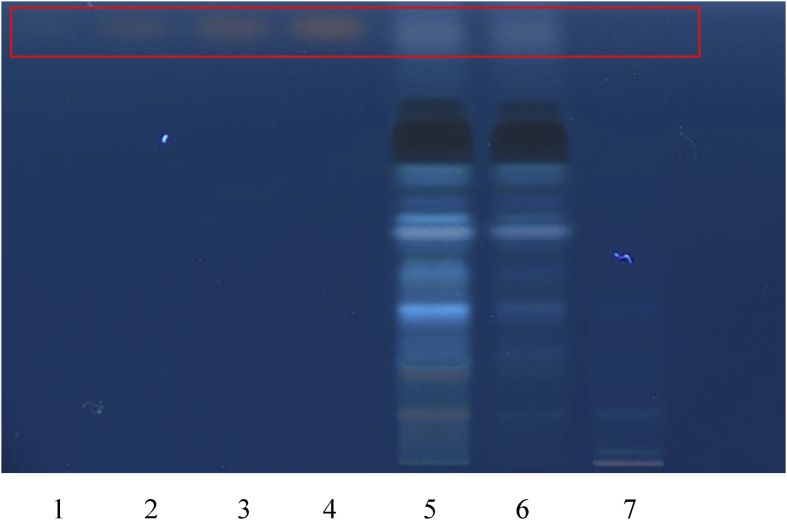
1–4: reference substance chrysophanol (5–20μL); 5: the RCM-107 formula in methanol; 6: the RCM-107 formula in ethanol; 7: Cassia seed sample.

According to the literature, cassia seed should contain chrysophanol (Priya et al., 2013). Samples and standards were developed on silica gel using the mobile phase ethyl acetate, methanol, water 80:20:10 (v/v/v) (Priya et al., 2013). Sulphuric acid (10%) reagent was used as the chemical derivatising agents and the results were evaluated under 366nm after derivatization. Chrysophanol gave an *Rf* value of 0.86 was used as an authentic standard (Fig. 7). However, both the RCM-107 formula and cassia seed samples showed no trace of chrysophanol under these conditions.

#### 3.2.6 Identification of alisol B acetate

**Fig. 8:**
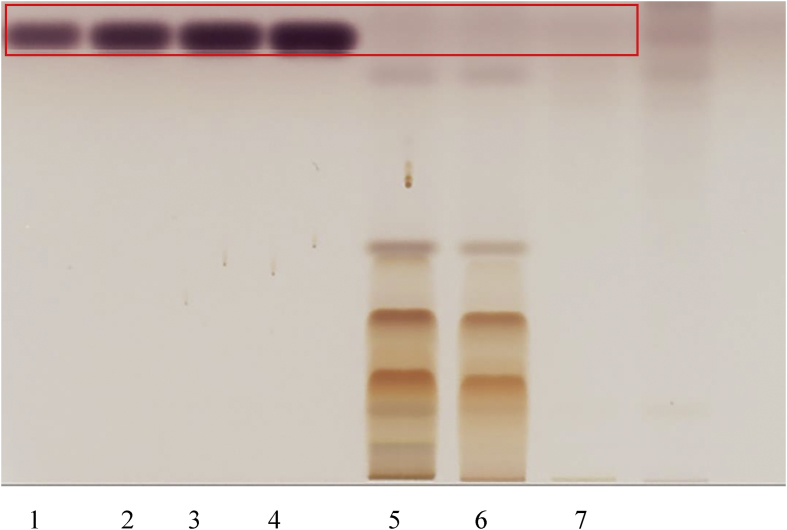
1–4: chemical reference substance alisol B acetate (5–20μL); 5: the RCM-107 formula in methanol; 6: the RCM-107 formula in ethanol; 7: *Alismatis rhiazoma* sample.

According to the literature, *Alismatis rhiazoma* should contain alisol B acetate (Wagner et al., 2016). Samples and standards were developed using the mobile phase chloroform, methanol, water 70:30:4 (v/v/v) (Wagner et al., 2016) and derivatized with *p*-anisaldehyde-sulfuric acid reagent (Wagner et al., 2016). Alisol B acetate gave an *Rf* value of 0.92 after derivatization under white light as the authentic standard (Fig. 8). However, no alisol B acetate was detected in the RCM-107 formula or *Alismatis rhiazoma* samples.

### 3.3 Molecular docking

In total seven ligands were selected for molecular docking, including the positive control, orlistat and the compounds identified in the HPTLC profiling experiments. Molecular docking was performed to study the interactions between the target (PDB code: 1ETH) and ligands (orlistat, EGCG, ECG, EC, caffeine, crocin and rutin).

A greater negative numerical value binding affinity indicates a firmer predicted binding in the ligand-target complex. The molecular docking results demonstrated that crocin had the highest binding affinity (-10 kcal/mol) to the 1ETH, followed by EC, EGCG, ECG (-9.6, -9.5 and -9.4 kcal/mol, respectively). On the other hand, rutin, the positive control orlistat and caffeine had the lowest binding energy (-8.7, -7.6 and -6.6 kcal/mol, respectively) compared to the others (Table 1). The majority of the selected ligands displayed stronger predicted binding affinity than the known lipase antagonist, orlistat.

**Table 1:** Binding affinity and hydrogen bonding of the ligands presented in HPTLC with pancreatic lipase (1ETH).

Herbal names	Ligands	Binding affinity (kcal/mol)	Conventional hydrogen bond (Amino acid)	Bond length (Å)
Green tea	EGCG	-9.5	TYR115(Chain A)	5.51
			HIS152(Chain A)	4.38
			ASP80(Chain A)	3.58
Green tea	ECG	-9.4	HIS152(Chain C)	4.41
			GLY77(Chain C)	3.07
Green tea	EC	-9.6	GLY77(Chain A)	3.17
Green tea	Caffeine	-6.6	ARG257(Chain C)	4.12
Lotus Leaf/*Sophorae flos*	Rutin	-8.7	GLU303(Chain C)	5.49
			VAL322(Chain C)	5.49
			ASN320 ((Chain C,2 BONDS)	4.61; 3.13
*Gardeniae fructus*	Crocin	-10	GLN220 (Chain C)	5.13
			GLN220 (Chain A)	4.94
			GLN22(Chain C)	5.66
			VAL21(Chain A)	3.93
			VAL21(Chain C)	4.11
			GLU188 (Chain C)	4.33
			ARG191(Chain A)	6.48
Lipase inhibitor	Orlistat	-7.6	GLY77(Chain A)	3.26
			HIS152(Chain A)	4.28
			ASP80(Chain A)	4.29
			SER153(Chain A)	4.64

**Fig. 9:**
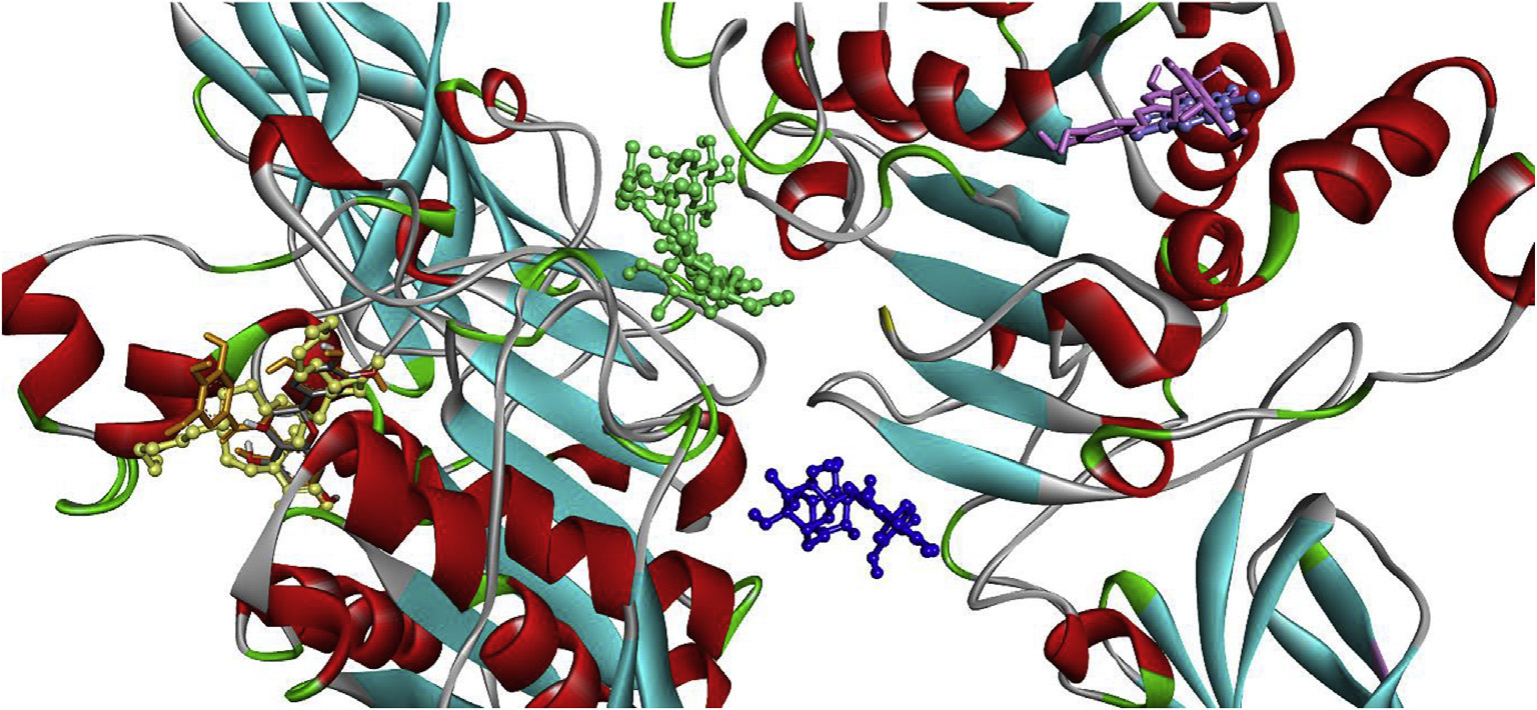
3D image showing the best binding mode of orlistat (yellow), EGCG (orange), EC (grey and red), crocin (green), rutin (blue), ECG (pink), caffeine (purple) within the porcine pancreatic lipase (PDB code: IETH).

There are two identical chains of 1ETH, the best binding mode of EGCG, EC and the orlistat located on the same chain of the target 1ETH (Fig. 9). Only 1 hydrogen bond interaction (3.17Å) was formed between the phenolic hydrogens and oxygen of EC (Sun et al., 2017) and the residue GLY77 while 3 hydrogen bonds with distances of 5.51 Å, 4.38 Å, and 3.58 Å, respectively; established with the side chains of TYR115, HIS152, and ASP80 for EGCG. In addition, orlistat formed 4 hydrogen bonds (3.26, 4.28, 4.29, 4.64 Å, respectively) with GLY77, HIS152, ASP80, SER153 (Table 1).

**Fig. 10:**
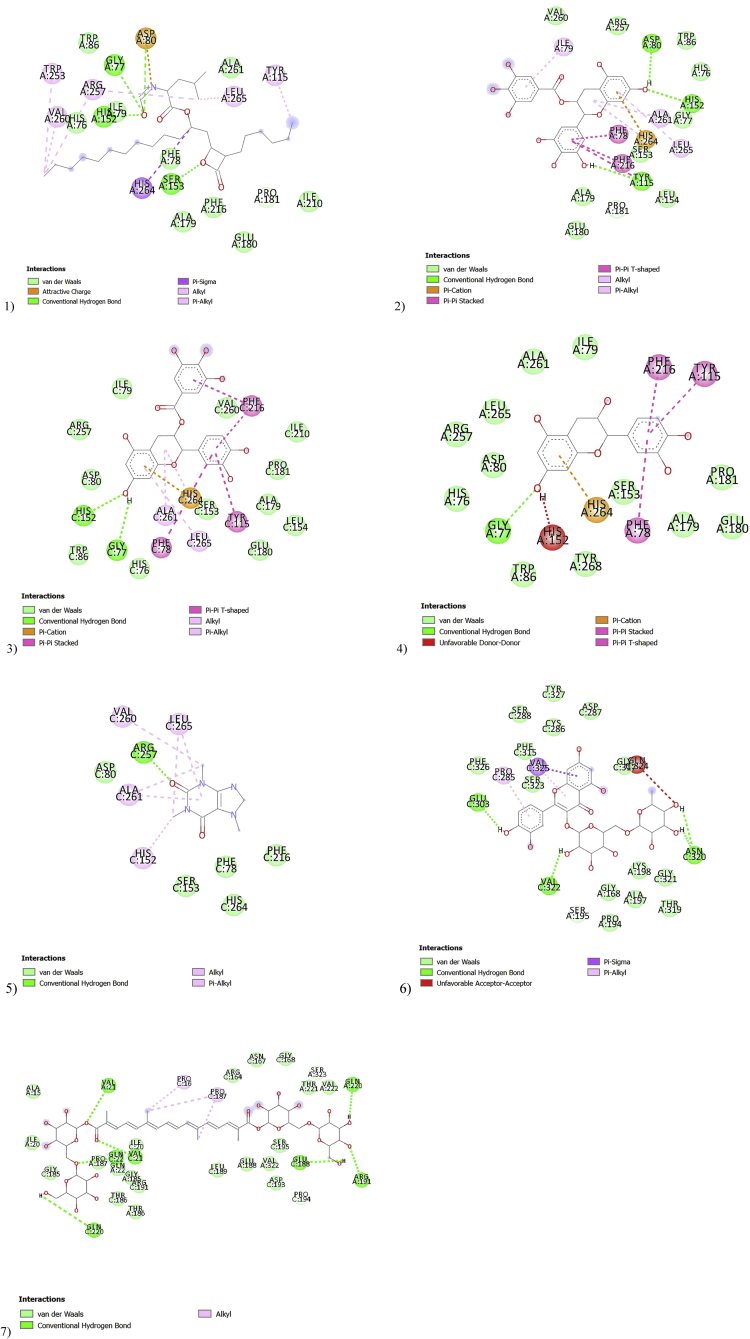
2D interactions between 1ETH and 1) orlistat, 2) EGCG, 3) ECG, 4) EC, 5) caffeine, 6) rutin, 7) crocin.

On the other chain of 1ETH, ECG was found to form hydrogen bonds with amino acid residues HIS152 and GLY77 (4.41 and3.07 Å, respectively). Caffeine formed 1 hydrogen bond with the residue ARG257 with a distance of 4.12 Å. In total four hydrogen bonds with amino acids GLU303, VAL322, ASN320 (2 bonds) (5.49, 5.49, 4.61, 3.13 Å, respectively) were displayed for the compound rutin while crocin established seven hydrogen bonds (5.13, 4.94, 5.66, 3.93, 4.11, 4.33, 6.48Å respectively) with GLN220 (1 bond each on each chain), GLN22, VAL21 (1bond each on each chain), GLU188, ARG191 of the 1ETH (Table 1). A diagram of the 2D interaction is illustrated in Fig. 10.

## 4 Discussion

To date, there are treatment regimens available for obesity, including lifestyle modifications (e.g. diet control and physical exercise) (Chen, 2016; National Health and Medical Research Council(NHMRC), 2013; Rodgers et al., 2012; Sun et al., 2016), pharmacotherapies (eg. orlistat, liraglutide and lorcaserin.) (Chen, 2016; Rodgers et al., 2012) and bariatric surgeries (e.g. restrictive procedures or malabsorptive procedures) (National Health and Medical Research Council (NHMRC), 2013). However, the treatment outcomes are usually disappointing as a result of failing to maintain lifestyle modifications (Sun et al., 2016) or dropping out from treatments due to disturbing side effects such as faecal incontinence and abdominal pain (Chen, 2016; Lucas and Kaplan-Machlis, 2001; Rodgers et al., 2012). Therefore, herbal supplements including medicinal plant extracts and Chinese herbal medicine as well and their associated natural products, have a long history of assisting obese individuals with weight management have become popular alternatives (Er, 2016; Esteghamati et al., 2015; Sun et al., 2016).

Anti-obesity mechanisms of herbal supplements include increasing energy expenditure, appetite suppression or blocking lipids absorption (Sun et al., 2016). The inhibition of lipase that interferes with the digestion of lipids and targets the gastrointestinal tract directly has been used as one of the promising strategies for weight reduction and management (Sun et al., 2016). Pancreatic (triacylglycerol) lipase is a key lipolytic enzyme secreted by the pancreas (Buchholz and Melzig, 2015). Inactivation of PL has been proven to have anti-obesity effects by forming a covalent bond with gastric and pancreatic lipase in the gut and stopping lipases turning dietary fat into absorbable monoglycerides and free fatty acids. Therefore weight loss can be induced due to a reduction of fat absorption and an attenuation of caloric absorption (Buchholz and Melzig, 2015).

Different enzymatic assays are available to detect the inhibitory potential of herbal supplements, natural plants or medicinal extracts (Buchholz and Melzig, 2015; Marrelli et al., 2014). In our pancreatic lipase inhibition study, a fluorescence-based assay was selected as it is sensitive than the methods such as spectrophotometry (Buchholz and Melzig, 2015). 4MUO, an oleate ester was used as the substrate and the presence of its highly-fluorescent hydrolysed product 4 MU was proportional to lipase activities (Buchholz and Melzig, 2015). Our data has shown that green tea exhibited the strongest inhibitory activity (89%). The RCM-107 formula, which exhibited an IC_50_ value of 7.17 ± 0.69 μg/mL, presented a slightly lower inhibition rate (88%) than green tea but this difference was statistically insignificant. The inhibition rate of the RCM-107 formula and other 3 single herbal granules, including green tea, *Sophorae flos* (80%) and *Gardeniae fructus* (74%) were higher than the known, irreversible lipase inhibitor orlistat (73%). However, only the RCM-107 formula and green tea are considered as statistically significant containing more potent inhibitors than orlistat at a concentration of 100 μg/mL. In addition, lotus leaf (68%), *Alismatis rhizome* (55%) and *Cassiae semen* (46%) showed less potent inhibitory effects than the above-mentioned herbs, but the reduction of lipase activities was statistically significant. On the other hand, *Poria* and *Plantaginis semen* displayed non-significant effects on suppressing pancreatic lipase, which may suggest that these 2 herbs have no inhibitory effects on lipase at this concentration (100 μg/mL). Overall, the strongest inhibitors of pancreatic lipase identified from the samples in this study were from green tea and the RCM-107 formula, slightly more potent than orlistat when they were assayed under the same conditions.

Several previous studies (Bustos et al., 2018; Cha et al., 2012; Sheng et al., 2006; Yuda et al., 2012) have identified the lipase inhibitory effects of polyphenols and flavonoids presented in the eight crude herbal ingredients from the RCM-107 formula. In a study by Cha et al. (2012), they investigated EGCG from green tea as a potent pancreatic lipase inhibitor with an IC_50_ of 1.8 ± 0.57 μM while 4MUO was also used as a substrate (Cha et al., 2012). ECG, one of the polyphenols that exhibited strong inhibitory action on pancreatic lipase had a IC_50_ value of 1.046 μg/mL (Yuda et al., 2012) while EC showed weak inhibitory effects with an IC_50_ of >500 μM (Cha et al., 2012). The flavonoid, rutin present in lotus leaf and *Sophorae flos* was suggested as a weak lipase inhibitor with an IC_50_ over 81.9 μM (Bustos et al., 2018). In addition, an *in vivo* study conducted by Sheng et al. (2006) reported the inhibitory effects of crocin on pancreatic lipase and showed its potential of decreasing lipase activates in a dose-dependent manner with an IC_50_ of 28.63 μM/L using emulsion as a substrate (Sheng et al., 2006). Quercetin has previously been reported as strong lipase inhibitor with an IC_50_ value of 21.5 μM ± 9.4 (Nakai et al., 2005). Moreover, Q3*O*G from lotus leaf (IC_50_ > 50 μg/mL) (Yuda et al., 2012), chrysophanol from *Cassia semen* (LIAO et al., 2007) and alisol B acetate from *Alismatis rhizoma* (Miao et al., 2017) has been mentioned in the literature for its possible lipase inhibition or weight reduction effects. Caffeine, on the other hand, has been reported to have no effects on pancreatic lipase (Yuda et al., 2012), which corresponds to our molecular docking results due to its lowest binding affinity with the target enzyme.

Ten compounds mentioned above were used as authentic standards to efficiently identify their presence in the RCM-107 formula and related single herbs via HPTLC profiling, which is an efficient, chemical profiling technique for the efficient analysis of small molecules (i.e. natural products) in biological matrices such as Chinese herbal extracts (Toniolo et al., 2014). It is rapid as well as cost-effective and the results can be directly displayed in the form of images in real-time. The authentic standards and samples are analysed in parallel, which allows the results to be evaluated in under 10 min by comparing the *Rf* of the authentic standard(s) to that either present or absent in the sample (Khokhlova and Zdoryk, 2016). HPTLC has also found applications in the fraudulence, adulteration, authentication, quality control of raw materials, food and herbal products (Khokhlova and Zdoryk, 2016). The presence of 6 standards in the RCM-107 formula and related single herbal granules, including flavonoids (rutin); polyphenols (EGCG, ECG, EC and caffeine) and crocin were identified using HPTLC. However, quercetin, Q3*O*G, alisol B acetate and chrysophanol were not detected (or potentially below the limits of detection of HPTLC) in the tested samples (Table 2). It is also likely that the absence of these desired compounds may be due to compound degradation during the extraction process (Xie et al., 2006). The oxidation process and stability of some compounds could be prone to changes in pH (Ramešová et al., 2012). For instance, alisol B acetate contains an unstable component epoxy heterocyclic, which initiates a ring-opening reaction and generates alisol A 24-acetate under acidic conditions (Zhang et al., 2014).

**Table 2:** HPTLC results sumamrising the presence or absence of active compounds present in target samples.

10 Standards	RCM-107 formula	Green tea	*Cassia semen*	*Sophorae flos*	Lotus leaf	*Gardeniae fructus*	*Alismatis rhizoma*
EGCG	✓	✓					
ECG	✓	✓					
EC	✓	✓					
Rutin	✓			✓			
Caffeine	✓	✓					
Crocin	✓					✓	
Alisol B acetate	X						X
Quercetin	X			✓			
Chrysphanol	X		X				
Q3*O*G	X				✓		

The six compounds identified in HPTLC were selected for molecular docking studies, aiming to elucidate the interactions and the best binding modes between selected ligands (i.e. small molecule compounds) and targets (i.e. macromolecules) (Adeniji et al., 2018). PyRx, an open-source molecular docking software was selected to conduct the computer-based analysis in order to obtain the optimal binding modes (bound conformation) and corresponding binding affinities (Dallakyan and Olson, 2015). In this study, the binding energy between ligands and the target protein as well as the profiles of protein-ligand interaction were predicted. Our docking results suggested that crocin, EC, EGCG, ECG exhibited stronger binding affinity in 1ETH than rutin, orlistat and caffeine, which may correlate with their biological activities (Adeniji et al., 2018).

Rutin and caffeine showed lower or no lipase inhibitory activities in previous enzyme assays, which is consistent with their predicted weaker binding affinity presented in the docking results (Bustos et al., 2018; Yuda et al., 2012). On the other hand, the known inhibitory potency of EGCG is inconsistent with the present molecular docking predictions. As mentioned earlier, EGCG is a more potent lipase inhibitor than ECG, EC and crocin according to their IC_50_ values reported in previous *in vitro* experiments (Cha et al., 2012; Sheng et al., 2006; Yuda et al., 2012), our docking results, however, demonstrated a weaker binding affinity of EGCG than crocin and EC. Thus, predicted docking binding affinity is likely to be only one of several factors that need to be taken into account in order to accurately predict inhibitory activity. Possible reasons that contribute to the weaker binding affinity of EGCG include: docking results can be affected by protonation state or proton exchange as a result of environmental differences (Hassan and Svajdlenka, 2017). In addition, other factors such as pH and molecular environment of particular compounds can affect the interactions between protein and ligands (Hassan and Svajdlenka, 2017).

**Fig. 11:**
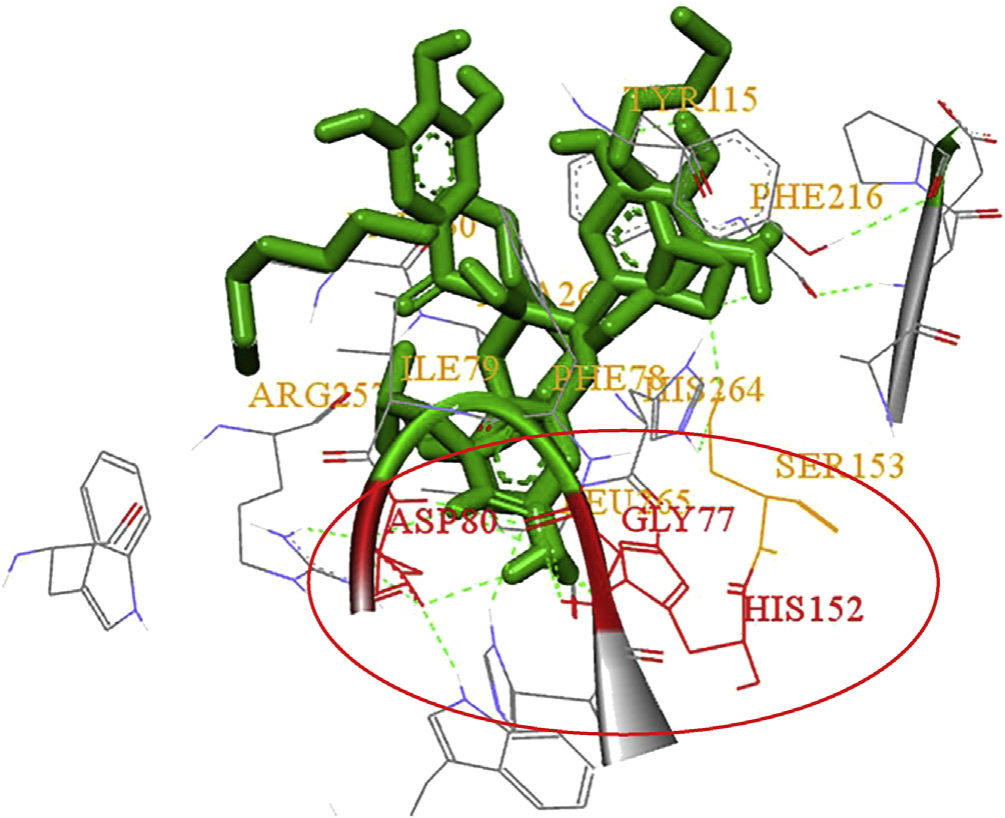
Amino acids interact with orlistat, EGCG and EC, their common residues have been circled and highlighted in red.

Inspection of the ligand-protein interactions in details revealed that HIS152, ASP80 and GLY77 were found as common residues that have formed hydrogen bonds with EGCG, EC and the known inhibitor orlistat at the same interaction site (Fig. 11), which may explain the role of the enzyme inhibition of EGCG and EC (Cha et al., 2012). Although ECG has also been found to interact with the same amino acid residues HIS152 and GLY77 as orlistat, its active site was on the other chain of 1ETH. In addition, rutin and crocin showed lipase inhibitory activities in the assay studies as mentioned earlier (Bustos et al., 2018; Sheng et al., 2006), and they were found to interact with amino acids at a site when distinct from that which bind the known inhibitor orlistat. Further experimental studies are warranted to fully elucidate the roles of the residues HIS152, ASP80 and GLY77 in their interactions with rutin and crocin.

## 5 Conclusion

In this study, the RCM-107 formula was found to be a potent lipase inhibitor, which could suppress lipid absorption and result in weight loss. The majority of ingredients from this formula also showed their lipase suppressing activities. The presence of 6 active weight-loss compounds has been effectively identified in the studied formula and related single granules. In addition, EGCG and EC have been found to interact with 1ETH at the same site as orlistat, forming hydrogen bonds with HIS152, ASP80 and GLY77 amino acid residues, which can be considered as markers of important areas in the ligand-binding site and may explain the details of their roles in inhibiting pancreatic lipase. Although further investigation is necessary for a more comprehensive understanding of the RCM-107 formula for weight loss, our preliminary results have provided new knowledge into the mechanisms and use of the studied herbal formula and single herbal granules in weight reduction. HPTLC was used as rapid chemical profiling tool (under 10 min) to identify either the absence or presence of the target compound in the formula. The quantification of the identified compounds along with linearity (Kumar et al., 2008), limits of detection (Kumar et al., 2008) will be carried out in future studies using either HPTLC and/or High performance liquid chromatography.

## Declarations

### Author contribution statement

Shiqi Luo: Conceived and designed the experiments; Performed the experiments; Analyzed and interpreted the data; Contributed reagents, materials, analysis tools or data; Wrote the paper.

George Binh Lenon, Harsharn Gill, Toan Linh Nguyen: Conceived and designed the experiments; Contributed reagents, materials, analysis tools or data; Wrote the paper.

Daniel Anthony Dias, Mingdi Li, Andrew Hung: Conceived and designed the experiments; Analyzed and interpreted the data; Contributed reagents, materials, analysis tools or data; Wrote the paper.

### Funding statement

This work was supported by Tong Lee Pty Ltd and the award of a School of Health and Biomedical Sciences Transitional Seed Funding Grant (RMIT University) Melbourne, Australia.

### Competing interest statement

The authors declare no conflict of interest.

### Additional information

The data used to support the findings of this study are available from the corresponding author upon request.
